# Heart Failure Disturbs Gut–Blood Barrier and Increases Plasma Trimethylamine, a Toxic Bacterial Metabolite

**DOI:** 10.3390/ijms21176161

**Published:** 2020-08-26

**Authors:** Adrian Drapala, Mateusz Szudzik, Dawid Chabowski, Izabella Mogilnicka, Kinga Jaworska, Katarzyna Kraszewska, Emilia Samborowska, Marcin Ufnal

**Affiliations:** 1 Laboratory of the Centre for Preclinical Research, Department of Experimental Physiology and Pathophysiology, Medical University of Warsaw, 02–097 Warsaw, Poland; adrapala@wum.edu.pl (A.D.); m.szudzik@igbzpan.pl (M.S.); chabowskidawid@gmail.com (D.C.); izabellamogilnicka@gmail.com (I.M.); kinga.jaworska@wum.edu.pl (K.J.); kjrutkowska@wp.pl (K.K.); 2 Mass Spectrometry Laboratory, Institute of Biochemistry and Biophysics, Polish Academy of Sciences, 02–106 Warsaw, Poland; emi.sambor@gmail.com

**Keywords:** bacterial metabolites, tight junctions, TMAO, intestinal barrier, leaky gut, cardiovascular disease

## Abstract

Trimethylamine (TMA) is a gut bacteria product oxidized by the liver to trimethylamine-*N*-oxide (TMAO). Clinical evidence suggests that cardiovascular disease is associated with increased plasma TMAO. However, little headway has been made in understanding this relationship on a mechanistic and molecular level. We investigated the mechanisms affecting plasma levels of TMAO in Spontaneously Hypertensive Heart Failure (SHHF) rats. Healthy Wistar Kyoto (WKY) and SHHF rats underwent metabolic, hemodynamic, histopathological and biochemical measurements, including tight junction proteins analysis. Stool, plasma and urine samples were evaluated for TMA and TMAO using ultra performance liquid chromatography-mass spectrometry. SHHF presented disturbances of the gut–blood barrier including reduced intestinal blood flow, decreased thickness of the colonic mucosa and alterations in tight junctions, such as claudin 1 and 3, and zonula occludens-1. This was associated with significantly higher plasma levels of TMA and TMAO and increased gut-to-blood penetration of TMA in SHHF compared to WKY. There was no difference in kidney function or liver oxidation of TMA to TMAO between WKY and SHHF. In conclusion, increased plasma TMAO in heart failure rats results from a perturbed gut–blood barrier and increased gut-to-blood passage of TMAO precursor, i.e., TMA. Increased gut-to-blood penetration of bacterial metabolites may be a marker and a mediator of cardiovascular pathology.

## 1. Introduction

Despite significant progress in diagnostics and treatments, mortality rates due to cardiovascular disease, such as heart failure, continue to increase [1]. While the pathogenesis of cardiovascular disease and its associated risk factors continues to puzzle researchers, accumulating evidence suggests that the gut microbiota plays an important role in human health and disease, including cardiovascular disease, and may contribute significantly to the onset and/or progression of cardiovascular disease [2]. Gut microbiota produce numerous biologically active metabolites, which can have either a positive or negative influence on the host. By crossing the intestinal wall, which forms the gut–blood barrier, these bacterial products can enter the systemic circulation and induce a plethora of physiological effects either at the point of entry or distant from it by traveling through the bloodstream. A growing body of evidence suggests that some bacteria-produced compounds may be considered mediators and/or markers of cardiovascular disease [3].

We have recently reported that trimethylamine (TMA), a product of the metabolism by gut bacteria of l-carnitine and choline, exerts toxic effects on the cardiovascular system. Specifically, we have shown that TMA elevates blood pressure [4], exerts cytotoxic effects on cardiomyocytes [5] and vascular smooth muscle cells [6], and disturbs the protein structure of cardiac LDH and albumin [5]. Furthermore, we have found that ageing compromises the gut–blood barrier, causing an increase in the penetration of TMA from the gut into the circulation [6].

TMA is absorbed from the large bowel and is in part oxidized by the liver to form trimethylamine N-oxide (TMAO). Elevated levels of TMAO in the plasma are associated with cardiovascular and cardiometabolic diseases [7,8,9,10,11]. Specifically, TMAO levels show a remarkably strong association with the development and progression of heart failure [10,12]. However, despite a growing body of evidence, mechanisms linking elevated levels of TMAO and poor prognosis in patients with heart failure remain incompletely understood [13,14].

The aim of the present study was to examine the effects of heart failure on the gut–blood barrier and to evaluate the changes in the levels of TMA and TMAO in the plasma associated with this condition.

## 2. Results

### 2.1. General Metabolic and Kidney Parameters

No significant differences in the average body weight and tibia length between Wistar Kyoto rats (WKY) and spontaneously hypertensive heart failure rats (SHHF) were observed. Similarly, there was no significant difference between the experimental groups in food intake. However, SHHF tended to have higher water intake and urine output. Additionally, SHHF showed significantly higher levels of sodium in the plasma. No significant differences were noted in the levels of potassium or creatinine between the groups. Data are summarized in Table 1.

### 2.2. Cardiovascular Parameters

SHHF showed hypertrophic cardiomyopathy with compromised systolic function. Specifically, in comparison to WKY, SHHF showed a significant increase in heart mass (80% increase), diameters of the intraventricular septum and posterior heart wall, and a decrease in stroke volume and ejection fraction (Table 1, Figure S1 in Supplementary Materials).

**Figure 1:**
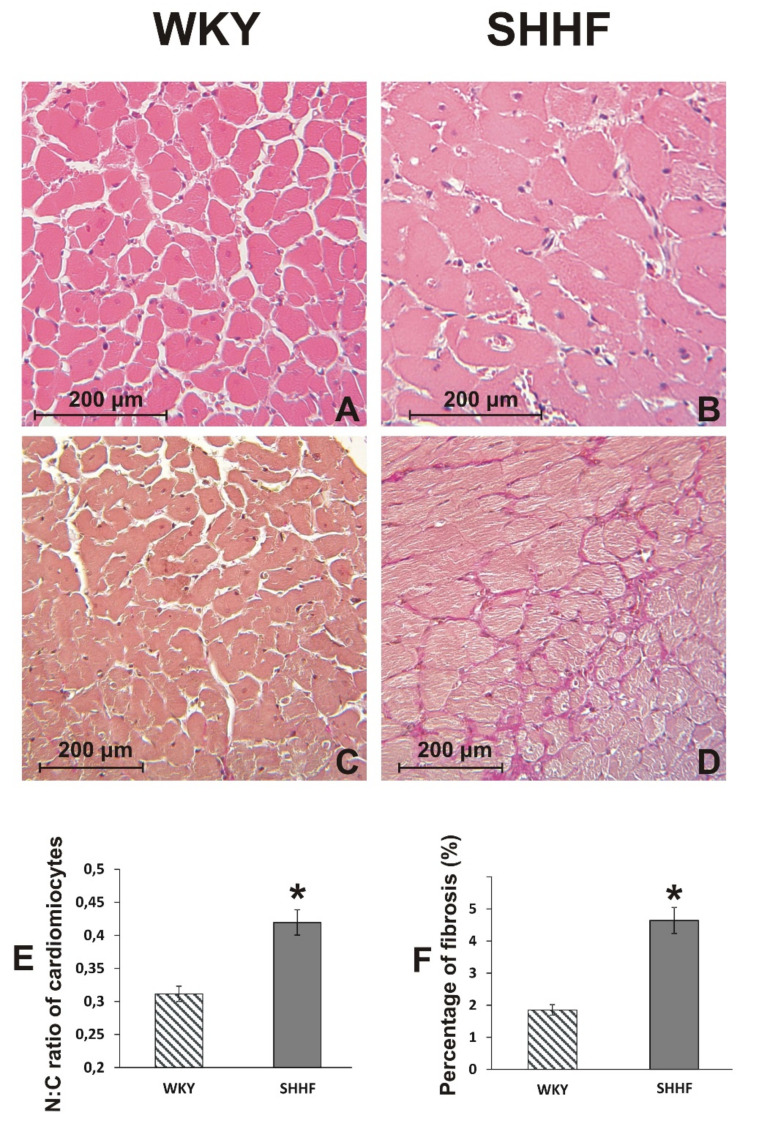
Histological image of the heart. (**A**,**B**) Cross section of cardiac muscle fibers stained with hematoxylin-eosin stain and viewed at a x10 magnification (lens). (**C**,**D**) Cross section of cardiac muscle fibers stained with van Gieson stain (for connective tissue fibres) and viewed at a ×10 magnification (objective lens). (**E**) The nuclear-cytoplasmic ratio of cardiomyocytes in WKY (*n* = 5) and SHHF (*n* = 5). (**F**) Percentage of myocardial fibrosis in WKY (*n* = 5) and SHHF (*n* = 5). Means ± SE, * *p* < 0.05 by *t*-test.

A histopathological picture of the heart suggests a transition from hypertrophic to dilated cardiomyopathy with a significantly increased ratio of nuclei size to cardiomyocyte size. Additionally, cardiac fibrosis was observed (Figure 1). SHHF had increased levels of NT-proBNP and vasopressin in the plasma. Finally, no pathological changes were found in the lungs of WKY rats, while, in contrast, SHHF showed a moderate interstitial edema (Figure S2 in Supplementary Materials).

### 2.3. Plasma Level of TMA and TMAO in WKY Rats and SHHF Rats with Heart Failure

SHHF showed significantly higher levels of TMA and TMAO in the plasma in comparison to WKY. Furthermore, the combined 24 h urine output of TMA and TMAO was higher in SHHF than in WKY. There was no significant difference in TMA levels in the colon content (stools) between WKY and SHHF rats. SHHF showed a significantly higher blood-to-stool concentration of TMA + TMAO, a marker of gut-to-blood permeability. In contrast, there was no significant difference between WKY and SHHF rats with respect to the TMAO/TMA ratio, an indicator of liver oxidation of TMA. All data are summarized in Table 2.

### 2.4. Intestines

There was significantly lower intestinal blood flow in SHHF than in WKY (Table 1, Figure S3 in Supplementary Materials).

**Figure 2:**
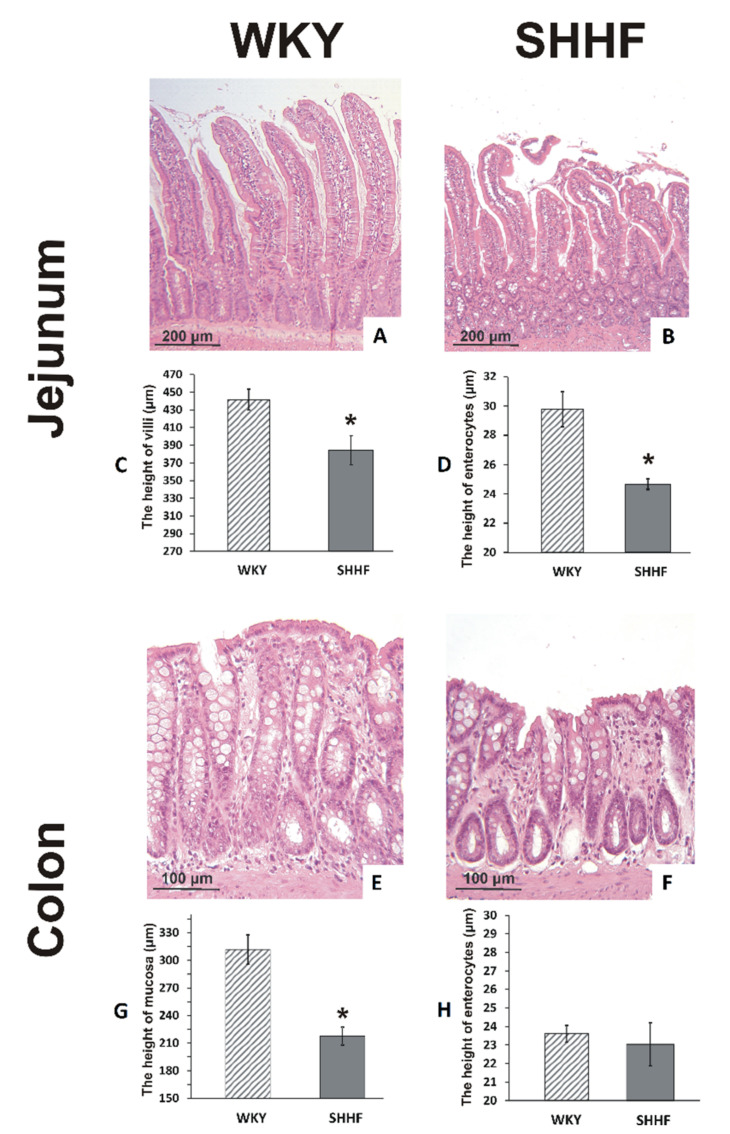
Histopathological evaluation of the intestine. (**A**,**B**) Histological images of the mucous membrane of the jejunum; (**E**,**F**) Histological images of the mucous membrane of the colon; stained with hematoxylin-eosin stain and viewed at a ×10 magnification (lens). (**C**) The height of the villi of the jejunum in WKY (*n* = 5) and SHHF (*n* = 6). (**D**) The height of enterocytes in the jejunum in WKY (*n* = 5) and SHHF (*n* = 6). (**G**) The height of the colonic mucosa in WKY (*n* = 5) and SHHF (*n* = 5). (**H**) The height of enterocytes in the colon of WKY (*n* = 5) and SHHF (*n* = 5). Means ± SE, * *p* < 0.05 by *t*-test.

Morphometric evaluation revealed a significantly lower height of the jejunum villi and the epithelium, and a significant reduction in the thickness of colonic mucosa in SHHF compared to WKY (Figure 2).

### 2.5. Tight Junction mRNA and Protein Levels

**Figure 3:**
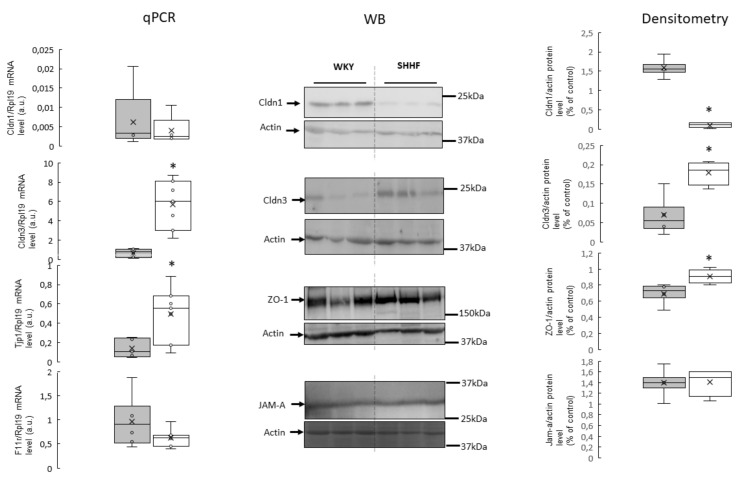
RT-qPCR analysis of claudin 1 (Cldn1), claudin 3 (Cldn3), zonula occludens-1 (ZO-1), and F11r transcript levels in the colon of WKY and SHHF rats (displays on histogram use arbitrary units), (means ± SD, *n* = 6–7). Western blot analysis of Cldn1, Cldn3, ZO-1 and junctional adhesion molecule A (JAM-A) protein levels from total protein extract prepared from the colon. A representative immunoblot is shown. Immunolabeled Cldn1, Cldn3, ZO-1, JAM-A and beta actin loading control bands were quantified using a Molecular Imager. The relative levels of the test proteins are plotted in arbitrary unit (means ± SD), grey bars—Wistar Kyoto rats, white bars—spontaneously hypertensive heart failure rats, * *p* < 0.05 by *t*-test.

**Figure 4:**
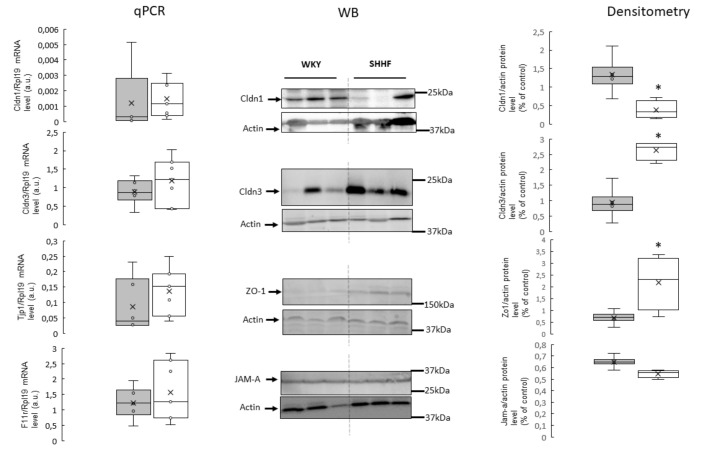
RT-qPCR analysis of claudin 1 (Cldn1), claudin 3 (Cldn3), zonula occludens-1 (ZO-1), and F11r transcript levels in the jejunum of the WKY and SHHF rats. (displays on histogram in arbitrary units (means ± SD, *n* = 6–7). Western blot analysis of Cldn1, Cldn3, ZO-1 and junctional adhesion molecule A (JAM-A) protein levels from total protein extract prepared from the jejunum. A representative immunoblot is shown. Immunolabeled Cldn1, Cldn3, ZO-1, JAM-A and beta actin loading control bands were quantified using a Molecular Imager. The relative levels of the test proteins are plotted in arbitrary unit (means ± SD), grey bars—Wistar Kyoto rats, white bars—spontaneously hypertensive heart failure rats, * *p* < 0.05 by *t*-test.

In the colon and jejunum, SHHF had significantly higher levels of claudin 3 (Cldn3) and zonula occludens-1 (ZO-1) proteins compared to WKY. In contrast, the levels of claudin 1 (Cldn1) protein were significantly lower in SHHF than in WKY. No significant difference in junctional adhesion molecule A (JAM-A) protein levels were found. In the colon, differences in protein levels were in close parallel with mRNA levels. In contrast, in the jejunum, there was no significant difference in mRNA levels, despite the above-mentioned differences in protein levels of Cldn1, Cldn3 and ZO-1 (Figure 3 and Figure 4, Figures S4 and S5 in Supplementary Materials).

## 3. Discussion

This study reports for the first time that heart failure disturbs the gut–blood barrier and increases gut-to-blood penetration of TMA, a toxic gut bacteria metabolite. Our study suggests that the increased plasma levels of TMAO in the SHHF rats are secondary to the increase in plasma TMA, a TMAO precursor. This scenario is a result of increased gut-to-blood penetration of TMA caused by disturbed function of the intestinal barrier in the rats with heart failure. This notion is based on the following findings: First, the SHHF had the same concentration of TMA in the colon content (which excludes differences in bacterial production of TMA) but a significantly higher level of TMA in the plasma. Hence, in comparison to WKY, SHHF showed a significantly higher blood-to-stool ratio of the bacterial metabolite concentration, a marker of gut–blood barrier permeability [15]. Second, the SHHF showed significant structural and functional disturbances in the intestinal wall in the form of reduced intestinal blood flow, decreased thickness of the colonic mucosa and disturbances in tight junction (TJ) proteins.

**Figure 5:**
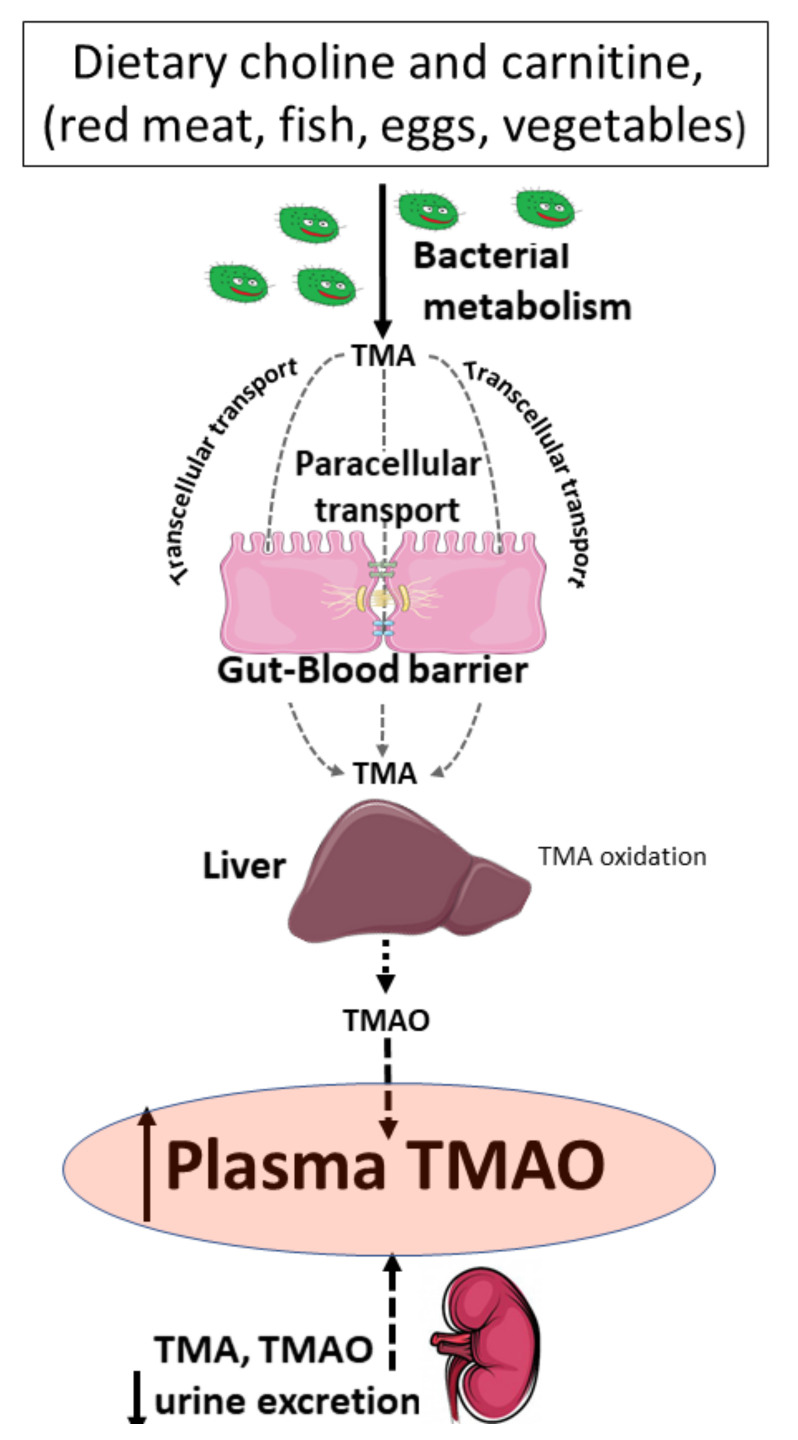
Factors affecting the levels of trimethylamine (TMA) and trimethylamine oxide (TMAO) in the plasma. Levels of TMA and TMAO in the plasma depend on the diet, bacterial metabolism, gut-to-blood penetration of TMA, liver oxidation of TMA to form TMAO, and the excretion of TMA and TMAO by the kidneys.

Several studies have shown that cardiovascular disease is associated with increased plasma levels of TMAO [10,11], and that TMAO levels are positively corelated with an increased risk of cardiovascular mortality [16,17]. However, the mechanisms responsible for the elevated levels of TMAO in the plasma are not clear. Plasma TMAO originates from gut bacteria-produced TMA that is subsequently oxidized in the liver to TMAO. The majority of TMA and TMAO is ultimately excreted in urine. Therefore, plasma TMAO levels depend on a three-way balance of gut bacterial activity, intestinal permeability to TMA, and liver and kidney function [18] (Figure 5). Interestingly, increased permeability in the gut has been suggested in patients with heart failure [19]. Reduced cardiac output and elevated systemic congestion results in the ischemia of the intestinal mucosa, which in turn may compromise and remodel the gut–blood barrier—a complex, multilayer cellular system of the intestinal wall.

TMA is easily soluble in both lipids and water. Therefore, both transcellular and paracellular pathways may be involved in the infiltration of TMA through the intestinal wall. As with other biological barriers, the permeability of the intestinal barrier is limited by its thickness [20]. In our study, SHHF had a significantly thinner colonic mucosa, which is a major site of bacterial metabolism, and showed a reduction in height of the jejunum villi and the epithelium. These alterations are likely the result of a significantly reduced intestinal blood supply in the rats with heart failure.

The gut–blood barrier is composed of many structural and functional elements. One important family of components that comprise the barrier are TJs, which consist of several transmembrane and cytoplasmic proteins, such as claudins, zonula occludens-1 (ZO-1), and junctional adhesion molecule A (JAM-A). TJs stabilize intercellular connections, maintain cell polarity, form specific apical domains and are involved in the regulation of key cellular functions, such as proliferation, differentiation and migration [21,22]. Regarding the levels of the TJs expression in intestinal diseases, the data are conflicting as both, increases and decreases in the expression of various TJs have been reported [21,22,23,24,25,26,27]. Such opposing findings may be the result of at least two factors. First, alternations in the expression of TJs may be secondary to the underlying intestinal diseases of various origins [21,22,24]. Second, some intestinal diseases may result from genetically determined alterations in the expression of TJs [28,29,30]. Moreover, it appears that changes in TJs may depend on the stage (severity) of the intestinal pathology [31,32,33].

In our study, in comparison to WKY, SHHF had significantly lower protein levels of Cldn1 but higher protein levels of Cldn3 and ZO-1. Based on our data, we propose that the altered expression of TJ proteins in SHHF were secondary to the disturbed intestinal blood flow and morphological changes in the intestines due to heart failure. To the best of our knowledge, this is the first study evaluating TJ proteins in the intestines of SHHF. Therefore, further studies are needed to elucidate the effect of the heart failure-dependent intestinal alterations on the expression of TJs.

The present study also provides evidence that SHHF have an increased leakage of TMA, a toxic gut bacteria product, from the gut into the systemic circulation, which suggest increased permeability of the gut in heart failure. These findings are in line with data published by Sandek et al., who showed increased permeability of the large intestine to sucralose in chronic heart failure patients [19,34]. Recently, we showed that hypertension [35] and ageing [6] are also associated with significant morphological and functional disturbances in the gut. They both increase the gut-to-blood leakage of TMA. These findings point to a critical role of the gut–blood barrier in controlling the levels of TMA and TMAO in the plasma.

TMA and TMAO are excreted primarily by the kidneys, and kidney failure is known to cause an increase in the levels of TMA and TMAO in the plasma [5,36]. In the present study, however, the SHHF did not present a significant decline in the kidney excretory function—i.e., creatinine and potassium plasma levels were similar in WKY and SHHF. Furthermore, SHHF showed greater 24-h urinary excretion of TMA and TMAO than WKY. These findings suggest that kidney failure was not a basis for increased plasma TMAO levels in SHHF.

Finally, plasma TMAO depends on liver function—i.e., oxidation of TMA to TMAO. However, we did not find a significant difference between the WKY and SHHF rats in terms of oxidation of TMA in the liver. Specifically, plasma TMAO/TMA ratio, an indicator of liver oxidation of TMA [37] was similar between the SHHF and WKY rats.

A limitation of our study is that we were not able to obtain enough portal blood from SHHF to measure the gut-to-portal blood permeability to TMA, as we have done in our previous studies of hypertensive rats [35]. Therefore, in the present study, to establish the permeability of the gut to TMA in the present study, we used an indirect method; i.e., we calculated a systemic blood-to-stool ratio of the concentration of TMA. The latter method was previously validated in our lab for other gut microbiota products [15].

**Figure 6:**
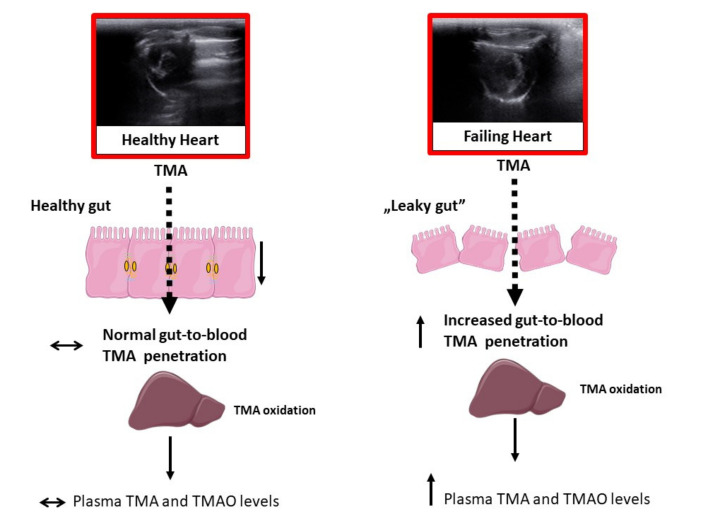
Proposed mechanism of increased plasma levels of trimethylamine oxide (TMAO) in rats with heart failure. Heart failure is associated with structural and functional alterations in the intestinal wall. This increases gut-to-blood penetration of trimethylamine (TMA) which is oxidized to TMAO by the liver.

In conclusion, heart failure is associated with significant structural and functional alterations in the intestinal wall. This results in elevated levels of TMA, a toxic gut bacteria metabolite, in the plasma and increased plasma levels of TMAO, a product of TMA oxidation in liver (Figure 6). Increased permeability of the gut-to-blood barrier to toxic bacterial metabolites, such as TMA, may be a marker and mediator of cardiovascular pathology.

## 4. Materials and Methods

The study was performed according to Directive 2010/63 EU on the protection of animals used for scientific purposes and approved by the I Local Bioethical Committee in Warsaw (permission:100/2016, approval date: 12/10/2016).

### 4.1. Animals

1-month-old, male, lean Spontaneously Hypertensive Heart Failure (SHHF/MccGmiCrl-*Lepr^cp^/*Crl, *n* = 8) rats were purchased from Charles River Laboratories (Reno, NV, USA). Age-matched Wistar Kyoto rats (WKY, *n* = 7) were obtained from the Central Laboratory for Experimental Animals, Medical University of Warsaw, Poland.

Rats were housed in groups of 2–3 animals, in polypropylene cages with environmental enrichment, 12 h light/12 h dark cycle, temperature 22–23 °C, humidity 45–55%, food and water ad libitum.

### 4.2. Metabolic and Hemodynamic Measurements

Two SHHF died before the experiment due to heart failure complications. Therefore, 6 SHHF and 7 WKY were included in the experiments. 12-month-old rats were housed in metabolic cages for 2 days to evaluate 24-h water and food balance and to collect urine for analysis. The next day, the rats were anaesthetized with urethane (1.5 g/kg b.w. i.p., Sigma-Aldrich, Poznan, Poland) and underwent an echocardiogram using a Samsung HM70 Ultrasound system equipped with a linear probe 5–13 MHz. The probe was placed on the shaved chest wall to obtain images from the right parasternal short axis. After the echo examination, the left femoral artery was cannulated with a polyurethane catheter for arterial blood pressure (ABP) recording and standard needle electrodes for ECG recordings were connected. The recordings began 40 min after the induction of anesthesia and 15 min after inserting the arterial catheter. After 10 min of ECG and ABP recordings, a Millar Mikro-Tip SPR-320 (2F) pressure catheter was inserted via the right common carotid artery and simultaneous left ventricle pressure and ABP recordings were performed. The catheter was connected to a Millar Transducer PCU-2000 Dual Channel Pressure Control Unit (Millar, Huston, TX, USA) and a Biopac MP 150 (Biopac Systems, Goleta, CA, USA). After removing the Millar catheter, the carotid artery was closed, and rats were subjected to measurements of intestinal blood flow (upper mesenteric vein blood flow) as described previously [38]. Briefly, after midline laparotomy, the intestines were lined outside the abdominal cavity and wrapped with moistened gauze to protect them from drying. The upper mesenteric vein, which collects blood from the colon, was separated from the surrounding tissue (for a distance of ca. 10 mm) to enable placement of a flow probe (ID 1 mm). Measurements were performed using a transit time ultrasound set-up which consists of a noncannulating acoustic (20 kHz) flow probe connected with a dedicated flowmeter (type T106, Transonic System Inc., Ithaca, NY, USA). The measurements were taken over a period of 10 min. After hemodynamic recordings, blood from the right ventricle of the heart was drawn and rats were euthanized by decapitation. The heart, lungs, kidneys, arteries, intestines and colon content (stools from the colon) were harvested for further analysis.

### 4.3. Stools, Plasma and Urine TMA, TMAO and General Biochemistry Evaluation

To evaluate the concentration of TMA and TMAO in stool samples, an 8–10 cm long segment of the middle colon was closed with sutures and removed. A sample of 0.5 mL of stool was collected from the removed colon, weighed and homogenized with 1 mL of 0.9% NaCl in a closed 2 mL Eppendorf tube by vortexing it for 5 min. Afterwards, the samples were centrifuged for 5 min at 5,000 RPM. From this supernatant, 1 mL was transferred to a fresh Eppendorf tube and centrifuged for an additional 5 min. All procedures were performed at 2–5 °C. The supernatant was collected into Eppendorf tubes and frozen at −20 °C.

Stool supernatant, plasma and urine concentrations of TMA and TMAO were evaluated using a Waters Acquity Ultra Performance Liquid Chromatograph (Waters, Illinois, MA, USA) coupled with a Waters TQ-S triple-quadrupole mass spectrometer (Waters, Manchester, UK). The mass spectrometer was operated in the multiple-reaction monitoring (MRM)–positive electrospray ionization (ESI) mode, as we previously described [39].

Plasma and urine sodium, potassium, creatinine and urea were analyzed using a Cobas 6000 analyzer (Roche Diagnostics, Indianapolis, IN, USA).

### 4.4. Histopathological Evaluation

Tissue sections were fixed in 10% buffered formalin, dehydrated using graded ethanol and xylene baths and embedded in paraffin wax. Sections of 3–4 μm were stained with a hematoxylin and eosin (HE) stain or a van Gieson stain (for connective tissue fibers). General histopathological examination was evaluated at a magnification of 10×, 40× and 100× (objective lens) and 10× (eyepiece) and photographic documentation was taken. Morphometric measurements were performed at magnification of 40× (objective lens).

### 4.5. ELISA Test

The following EIAab Kits (Wuhan EIAab Science Co. Ltd., Wuhan, Hubei, China) were used for the evaluation: NT-proBNP (cat. no. E0485r), aldosterone (cat. no. E0911Ge), vasopressin (cat. no. E1139Ge). All protocols were performed according to the standard protocol by ELISA Kit Operating Instruction. The absorbance intensity was measured at 450 nm with a Multiskan microplate reader (Thermo Fisher Scientific, Waltham, MA, USA). All experiments were performed in duplicates.

### 4.6. RNA Isolation and RT-qPCR

Total cellular RNA was extracted from the jejunum and colon (approximately 15 mg of wet tissue) using a Trizol reagent (Invitrogen, Carlsbad, CA, USA) according to the manufacturer’s protocol. Next, 1.5 micrograms of total DNAse-treated RNA was reverse transcribed using an Iscript^®^ (Bio-Rad, Hercules, CA, USA). Real-time quantitative PCR analysis was performed via a Bio-Rad real time system using gene-specific primer pairs shown in Table S1 in Supplementary Materials. The amplified products were detected using an iTaq^®^ Universal SYBR Green Supermix, (Bio-rad, Hercules, CA, USA). To confirm the specificity of amplification, the PCR products were subjected to a melting curve analysis and agarose gel electrophoresis. Bio-Rad CFX Maestro Software (Hercules, CA, USA) was used for data analysis. Transcript levels were normalized relative to the *Rpl19* reference gene, which was selected from four different housekeeping genes using NormFinder software (version 0.953, MOMA, Aarhus, Denmark).

### 4.7. Protein Extraction and Immunoblotting Analysis

For the analysis of target proteins, total protein extracts were prepared from the jejunum and colon. In short, frozen jejunum or colon samples were suspended in a histidine-sucrose buffer (30 mM histidine, 250 mM sucrose, 2 mM EDTA, proteases inhibitors, PMSF, pH 7.4), homogenized, centrifuged (10,000 RCF, 10 min, 4 °C). After removing the supernatant, 150 μL of lysis buffer (20 mM HEPES pH 7.4, 150 mM NaCl, 1 mM EDTA, 2% Triton-X, proteases inhibitors) was added to the pellet and resuspended by vortexing. The supernatant was separated for protein concentration analysis using a Bradford Protein Assay (Bio-Rad, Hercules, CA, USA). For all Western blot analyses, a 2× Laemmli sample buffer was added to samples. To determinate the levels of claudin 1 (Cldn1), claudin 3 (Cldn3), zonula occludens-1 (ZO-1) and junctional adhesion molecule A (JAM-A), all samples were resolved by electrophoresis on 12% SDS/PAGE gels for Cldn1, Cldn3, JAMA-A and 8% SDS/PAGE gels for ZO-1.

Resolved proteins were transferred onto PVDF membranes (Bio-Rad, Hercules, CA, USA), blocked using skim milk and incubated with primary and secondary antibodies (Table S2 in Supplementary Materials). For quantitative analysis of protein content, reactive bands were quantified relative to those of actin using a ChemiDoc MP Imaging System with a Quantity One software (Bio-Rad, Hercules, CA, USA).

### 4.8. Data Analysis and Statistics

Mean ABP, diastolic ABP, systolic ABP, and heart rate were calculated from the ABP tracing. Left ventricular end-diastolic pressure (LVEDP), maximal slope of systolic pressure increment (+dP/dt) and diastolic pressure decrement (-dP/dt) were calculated from the left ventricle blood pressure tracing using AcqKnowledge Biopac software (Biopac Systems, Goleta, CA, USA).

Differences between the groups were evaluated using an independent-samples *t*-test. A value of two-sided *p* < 0.05 was considered significant. Analyses were conducted using a Dell Statistica, version 13 (Dell Inc., Tulsa, OK, USA).

## Figures and Tables

**Table 1:** Metabolic and cardiovascular parameters in healthy Wistar Kyoto rats (WKY), and spontaneously hypertensive heart failure rats (SHHF).

Group/ Parameter	WKY	SHHF
**Energy and water balance**		
Body mass (g)	427.4 ± 12.9	469.8 ± 25.1
Tibia length (cm)	3.58 ± 0.15	3.82 ± 0.12
24 h food intake (g)	23.43 ± 1.24	21.94 ± 1.44
24 h water intake (mL)	33.3 ± 1.8	37.6 ± 2.1
24 h stool output (g)	9.5 ± 0.8	11.2 ± 1.8
24 h urine output (g)	14.6 ± 2.1	15.8 ± 0.7
**Electrolyte balance**		
Plasma sodium (mM)	137.45 ± 0.87	143.97 ± 1.3 *
Plasma potassium (mM)	4.47 ± 0.13	4.78 ± 0.18
Plasma creatinine (mM)	0.07 ± 0.007	0.07 ± 0.002
**Hormones**		
Aldosterone (ng/mL)	8.13 ± 1.01	8.16 ± 1.34
Vasopressin (pg/mL)	1.516.9 ± 132.1	3.222.4 ± 345.4 *
NT-proBNP (pg/mL)	30.98 ± 2.70	47.60 ± 1.31 *
**Heart mass**		
Heart mass	1.07 ± 0.04	1.85 ± 0.16 *
**Arterial blood pressure and heart rate**		
Systolic (mmHg)	106.9 ± 4.9	109.4 ± 4.9
Diastolic (mmHg)	66.7 ± 4.1	80.1 ± 4.5 *
HR (beats/min)	309 ± 22	312 ± 35
**Echocardiographic parameters**		
LV EDV (mL)	0.57 ± 0.03	0.28 ± 0.04 *
LV ESV (mL)	0.13 ± 0.03	0.11 ± 0.03
IVSs (cm)	0.25 ± 0.02	0.40 ± 0.01 *
IVSd (cm)	0.20 ± 0.01	0.29 ± 0.02 *
LVPWs (cm)	0.29 ± 0.02	0.40 ± 0.03 *
LVPWd (cm)	0.20 ± 0.01	0.30 ± 0.02 *
SV (mL)	0.44 ± 0.02	0.18 ± 0.03 *
EF (%)	76.9 ± 3.5	65.0 ± 3.9 *
**Left ventricle hemodynamic parameters (direct measurements)**		
LVEDP (mmHg)	5.23 ± 0.63	4.73 ± 0.67
dP/dt (mmHg/ms)	7.02 ± 0.36	6.28 ± 0.08
-dP/dt (mmHg/ms)	3.53 ± 0.45	2.43 ± 0.10
**ECG**		
RR (ms)	200.2 ± 16.6	207.7 ± 7.2
QT (ms)	50.0 ± 1.5	63.8 ± 3.9 *
QTc (ms)	112.5 ± 3.4	141.4 ± 8.2 *
PR (ms)	52.5 ± 2.2	57.9 ± 2.1
QRS width (ms)	20.6 ± 1.2	28.9 ± 2.1 *
QRS amplitude (mV)	2.61 ± 0.40	1.62 ± 0.17 *
**Intestinal blood flow**		
Intestinal blood flow (mL/min)	8.56 ± 1.35	5.28 ± 0.75 *

LVEDV—left ventricle end diastolic volume, LVESV—left ventricle end systolic volume, SV—stroke volume, EF—ejection fraction, IVSs—interventricular septum diameter during systole, posterior wall of the left ventricle during systole. LVEDP—pressure in the left ventricle during the end of diastole measured directly with a catheter, +dP/dt—maximal slope of systolic ventricular pressure increment,—dP/dt—maximal slope of diastolic ventricular pressure decrement. Values are means, ± SE. * *p* < 0.05 vs. WKY.

**Table 2:** TMA/TMAO balance in healthy Wistar Kyoto rats (WKY) and spontaneously hypertensive heart failure rats (SHHF).

Group/ Parameter	WKY	SHHF
Stool TMA (µM)	375.3 ± 25.7	304.1 ± 20.8
Stool TMAO (µM)	LQQ	LQQ
Plasma TMA (µM)	97.42 ± 6.7	153.0 ± 20.3 *
Plasma TMAO (µM)	4.57 ± 0.39	6.67 ± 0.69 *
Liver TMA oxidation (plasma TMAO/TMA ratio)	0.049 ± 0.005	0.048 ± 0.006
Gut permeability (plasma TMA+TMAO/stool TMA)	0.28 ± 0.03	0.54 ± 0.08 *
Urine TMA (µM)	594.20 ± 32.73	731.41 ± 98.99 *
Urine TMAO (µM)	432.63 ± 53.61	667.61 ± 64.13 *
24 h urine output TMA + TMAO (µmol/24 h)	16.07 ± 1.98	21.09 ± 1.55 *

TMA—trimethylamine, TMAO—trimethylamine N-oxide, LQQ—below the limit of quantification. Values are means, ± SE. * *p* < 0.05 vs. WKY.

## Supplementary Materials

Supplementary materials can be found at https://www.mdpi.com/1422-0067/21/17/6161/s1. Figure S1. (**A**) Electrocardiograph and (**B**) echocardiograph recordings in healthy Wistar Kyoto rats (WKY) and spontaneously hypertensive-heart failure rats (SHHF); Figure S2. Histological images of the lungs; Figure S3. Recordings of arterial blood pressure (BP), heart rate (HR) and intestinal blood flow (IBF) in healthy Wistar Kyoto rats (WKY) and spontaneously hypertensive heart failure rats (SHHF); Figure S4. Uncropped immunoblots from Figure 6; Figure S5. Uncropped immunoblots from Figure 4; Table S1. List of oligonucleotide primers used for RT-qPCR; Table S2. List of antibodies used for Western blot analyses.

## Abbreviations

TMA	trimethylamine
TMAO	Trimethylamine N-oxide
WKY	Wistar Kyoto rats
SHHF	Spontaneously Hypertensive-Heart-Failure rats
Cldn1	claudin 1
Cldn3	claudin 3
ZO-1	zonula occludens-1
JAM-A	junctional adhesion molecule A
ABP	Arterial blood pressure
LVEDP	Left ventricular end-diastolic pressure

## References

[1] Mensah G.A., Roth G.A., Fuster V. (2019). The Global Burden of Cardiovascular Diseases and Risk Factors. J. Am. Coll. Cardiol..

[2] Ahmadmehrabi S., Tang W.W. (2017). Gut microbiome and its role in cardiovascular diseases. Curr. Opin. Cardiol..

[3] Landfald B., Valeur J., Berstad A., Raa J. (2017). Microbial trimethylamine-N-oxide as a disease marker: Something fishy?. Microb. Ecol. Health Dis..

[4] Jaworska K., Bielinska K., Gawrys-Kopczynska M., Ufnal M. (2019). TMA (trimethylamine), but not its oxide TMAO (trimethylamine-oxide), exerts haemodynamic effects: Implications for interpretation of cardiovascular actions of gut microbiome. Cardiovasc. Res..

[5] Jaworska K., Hering D., Mosieniak G., Bielak-Zmijewska A., Pilz M., Konwerski M., Gasecka A., Kaplon-Cieslicka A., Filipiak K., Sikora E. (2019). TMA, A Forgotten Uremic Toxin, but Not TMAO, Is Involved in Cardiovascular Pathology. Toxins.

[6] Jaworska K., Konop M., Hutsch T., Perlejewski K., Radkowski M., Grochowska M., Bielak-Zmijewska A., Mosieniak G., Sikora E., Ufnal M. (2019). TMA but not TMAO increases with age in rat plasma and affects smooth muscle cells viability. J. Gerontol. A Biol. Sci. Med. Sci..

[7] Li X.S., Obeid S., Klingenberg R., Gencer B., Mach F., Raber L., Windecker S., Rodondi N., Nanchen D., Muller O. (2017). Gut microbiota-dependent trimethylamine N-oxide in acute coronary syndromes: A prognostic marker for incident cardiovascular events beyond traditional risk factors. Eur. Heart J..

[8] Qi J., You T., Li J., Pan T., Xiang L., Han Y., Zhu L. (2018). Circulating trimethylamine N-oxide and the risk of cardiovascular diseases: A systematic review and meta-analysis of 11 prospective cohort studies. J. Cell. Mol. Med..

[9] Senthong V., Wang Z., Fan Y., Wu Y., Hazen S.L., Tang W.H. (2016). Trimethylamine N-Oxide and Mortality Risk in Patients With Peripheral Artery Disease. J. Am. Heart Assoc..

[10] Tang W.H., Wang Z., Levison B.S., Koeth R.A., Britt E.B., Fu X., Wu Y., Hazen S.L. (2013). Intestinal microbial metabolism of phosphatidylcholine and cardiovascular risk. N. Engl. J. Med..

[11] Troseid M., Ueland T., Hov J.R., Svardal A., Gregersen I., Dahl C.P., Aakhus S., Gude E., Bjorndal B., Halvorsen B. (2015). Microbiota-dependent metabolite trimethylamine-N-oxide is associated with disease severity and survival of patients with chronic heart failure. J. Intern. Med..

[12] Tang W.H., Wang Z., Fan Y., Levison B., Hazen J.E., Donahue L.M., Wu Y., Hazen S.L. (2014). Prognostic value of elevated levels of intestinal microbe-generated metabolite trimethylamine-N-oxide in patients with heart failure: Refining the gut hypothesis. J. Am. Coll. Cardiol..

[13] Samulak J.J., Sawicka A.K., Samborowska E., Olek R.A. (2019). Plasma trimethylamine-N-oxide following cessation of L-carnitine supplementation in healthy aged women. Nutrients.

[14] Querio G., Antoniotti S., Levi R., Gallo M.P. (2019). Trimethylamine N-Oxide Does Not Impact Viability, ROS Production, and Mitochondrial Membrane Potential of Adult Rat Cardiomyocytes. Int. J. Mol. Sci..

[15] Jaworska K., Konop M., Bielinska K., Hutsch T., Dziekiewicz M., Banaszkiewicz A., Ufnal M. (2019). Inflammatory bowel disease is associated with increased gut-to-blood penetration of short-chain fatty acids: A new, non-invasive marker of a functional intestinal lesion. Exp. Physiol..

[16] Suzuki T., Heaney L.M., Bhandari S.S., Jones D.J., Ng L.L. (2016). Trimethylamine N-oxide and prognosis in acute heart failure. Heart Fail. Clin..

[17] Suzuki T., Heaney L.M., Jones D.J., Ng L.L. (2017). Trimethylamine N-oxide and Risk Stratification after Acute Myocardial Infarction. Clin. Chem..

[18] Ufnal M., Zadlo A., Ostaszewski R. (2015). TMAO: A small molecule of great expectations. Nutrition.

[19] Sandek A., Bauditz J., Swidsinski A., Buhner S., Weber-Eibel J., von Haehling S., Schroedl W., Karhausen T., Doehner W., Rauchhaus M. (2007). Altered intestinal function in patients with chronic heart failure. J. Am. Coll. Cardiol..

[20] Farhadi A., Banan A., Fields J., Keshavarzian A. (2003). Intestinal barrier: An interface between health and disease. J. Gastroenterol. Hepatol..

[21] Bhat A.A., Uppada S., Achkar I.W., Hashem S., Yadav S.K., Shanmugakonar M., Al-Naemi H.A., Haris M., Uddin S. (2019). Tight junction proteins and signaling pathways in cancer and inflammation: A functional crosstalk. Front. Physiol..

[22] Wong M., Ganapathy A.S., Suchanec E., Laidler L., Ma T., Nighot P. (2019). Intestinal epithelial tight junction barrier regulation by autophagy-related protein atg6/beclin 1. Am. J. Physiol. Cell Physiol..

[23] Dhawan P., Singh A.B., Deane N.G., No Y., Shiou S.-R., Schmidt C., Neff J., Washington M.K., Beauchamp R.D. (2005). Claudin-1 regulates cellular transformation and metastatic behavior in colon cancer. J. Clin. Investig..

[24] Kinugasa T., Akagi Y., Yoshida T., Ryu Y., Shiratuchi I., Ishibashi N., Shirouzu K. (2010). Increased claudin-1 protein expression contributes to tumorigenesis in ulcerative colitis-associated colorectal cancer. Anticancer Res..

[25] Prasad S., Mingrino R., Kaukinen K., Hayes K.L., Powell R.M., MacDonald T.T., Collins J.E. (2005). Inflammatory processes have differential effects on claudins 2, 3 and 4 in colonic epithelial cells. Lab. Investig..

[26] Turner J.R. (2009). Intestinal mucosal barrier function in health and disease. Nat. Rev. Immunol..

[27] Gröne J., Weber B., Staub E., Heinze M., Klaman I., Pilarsky C., Hermann K., Castanos-Velez E., Röpcke S., Mann B. (2007). Differential expression of genes encoding tight junction proteins in colorectal cancer: Frequent dysregulation of claudin-1,-8 and-12. Int. J. Colorectal Dis..

[28] Tamura A., Kitano Y., Hata M., Katsuno T., Moriwaki K., Sasaki H., Hayashi H., Suzuki Y., Noda T., Furuse M. (2008). Megaintestine in claudin-15–deficient mice. Gastroenterology.

[29] Xing T., Benderman L.J., Sabu S., Parker J., Yang J., Lu Q., Ding L., Chen Y.-H. (2019). Hepatology, Tight junction protein Claudin-7 is essential for intestinal epithelial stem cell self-renewal and differentiation. Cell. Mol. Gastroenterol..

[30] Xing T., Camacho Salazar R., Chen Y.-H. (2017). Animal models for studying epithelial barriers in neonatal necrotizing enterocolitis, inflammatory bowel disease and colorectal cancer. Tissue Barriers.

[31] Kirschner N., Poetzl C., von den Driesch P., Wladykowski E., Moll I., Behne M.J., Brandner J.M. (2009). Alteration of tight junction proteins is an early event in psoriasis: Putative involvement of proinflammatory cytokines. Am. J. Pathol..

[32] Xu C.-M., Li X.-M., Qin B.-z., Liu B. (2016). Effect of tight junction protein of intestinal epithelium and permeability of colonic mucosa in pathogenesis of injured colonic barrier during chronic recovery stage of rats with inflammatory bowel disease. Asian Pac. J. Trop. Med..

[33] Zeissig S., Bürgel N., Günzel D., Richter J., Mankertz J., Wahnschaffe U., Kroesen A.J., Zeitz M., Fromm M., Schulzke J.D. (2007). Changes in expression and distribution of claudin 2, 5 and 8 lead to discontinuous tight junctions and barrier dysfunction in active Crohn’s disease. Gut.

[34] Sandek A., Swidsinski A., Schroedl W., Watson A., Valentova M., Herrmann R., Scherbakov N., Cramer L., Rauchhaus M., Grosse-Herrenthey A. (2014). Intestinal blood flow in patients with chronic heart failure: A link with bacterial growth, gastrointestinal symptoms, and cachexia. J. Am. Coll. Cardiol..

[35] Jaworska K., Huc T., Samborowska E., Dobrowolski L., Bielinska K., Gawlak M., Ufnal M. (2017). Hypertension in rats is associated with an increased permeability of the colon to TMA, a gut bacteria metabolite. PLoS ONE.

[36] Wills M.R., Savory J. (1981). Biochemistry of renal failure. Ann. Clin. Lab. Sci..

[37] Mackay R.J., McEntyre C.J., Henderson C., Lever M., George P.M. (2011). Trimethylaminuria: Causes and diagnosis of a socially distressing condition. Clin. Biochem. Rev..

[38] Tomasova L., Dobrowolski L., Jurkowska H., Wrobel M., Huc T., Ondrias K., Ostaszewski R., Ufnal M. (2016). Intracolonic hydrogen sulfide lowers blood pressure in rats. Nitric Oxide: Biol. Chem..

[39] Jaworska K., Huc T., Gawrys M., Onyszkiewicz M., Samborowska E., Ufnal M. (2018). An In Vivo Method for Evaluating the Gut-Blood Barrier and Liver Metabolism of Microbiota Products. J. Vis. Exp..

