# Supplementary material for: Heart Failure Disturbs Gut–Blood Barrier and Increases Plasma Trimethylamine, a Toxic Bacterial Metabolite
Source: Int J Mol Sci. 2020 Aug 26;21(17):6161. doi: 10.3390/ijms21176161 (PMC7504565; doi:10.3390/ijms21176161)
Supplement: Supplementary file 1 [file ijms-21-06161-s001.pdf]

SUPPLEMENTARY MATERIAL

Supplementary Figures

Figure S1. (A) Electrocardiograph and (B) echocardiograph recordings in healthy Wistar Kyoto rats (WKY) and spontaneously-hypertensive-heart failure rats (SHHF). Comparing both electrocardiograms, the main differences visible are QTc, QRS prolongation and depression of ST segment in the SHHF rats. These findings reflect elecrocardiographic expression of left ventricular hypertrophy (see also Table 1). A mixed hypertrophy is responsible for the echocardiographic changes in the SHHF rats (see also Table 1).

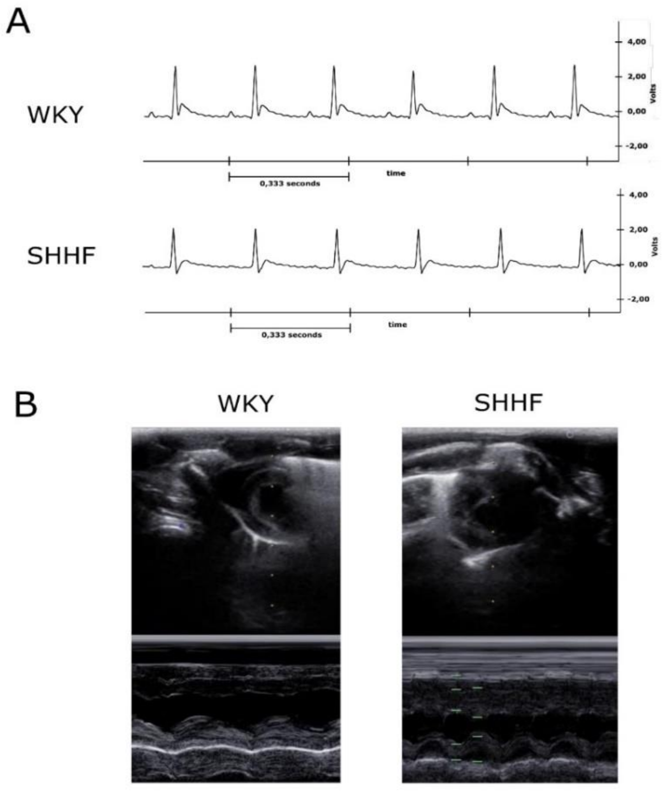

Figure S2. Histological images of the lungs. A, B – Lung parenchyma stained with hematoxylin-eosin and viewed at a magnification of x10 (lens). Interstitial edema, thickening and passive congestion of the alveolar walls and interalveolar septum is evident in SHHF.

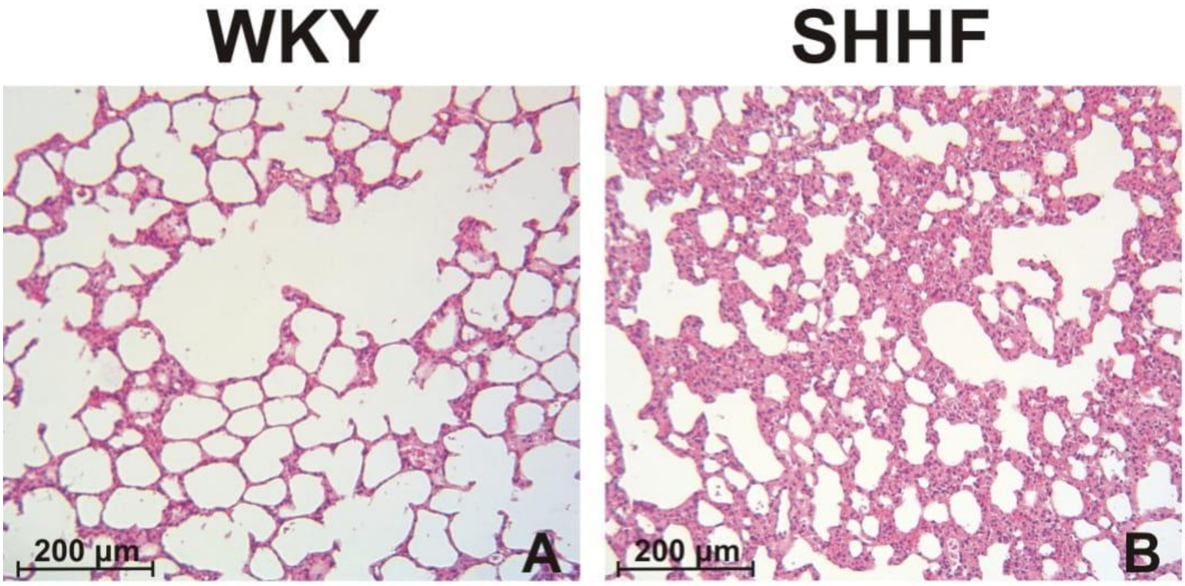

Figure S3. Recordings of arterial blood pressure (BP), heart rate (HR) and intestinal blood flow (IBF) in healthy Wistar Kyoto rats (WKY) and spontaneously hypertensive heart failure rats (SHHF).

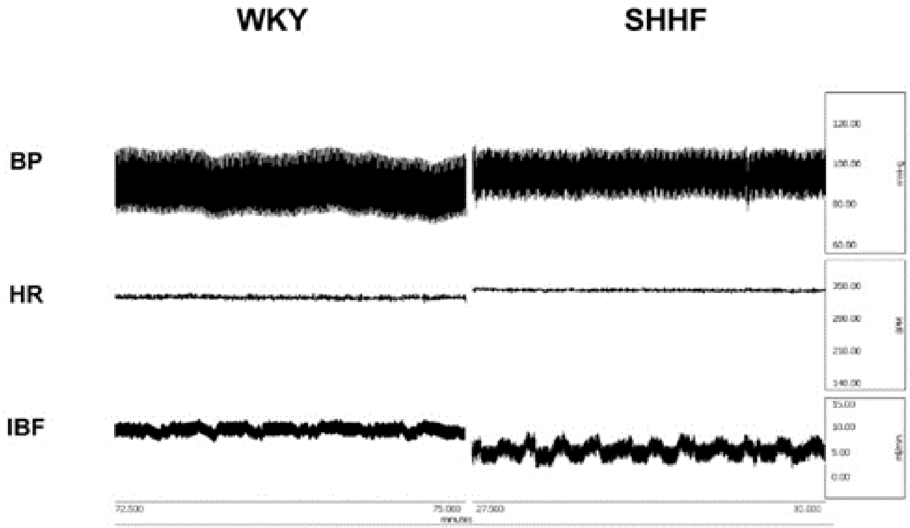

Figure S4. **Uncropped immunoblots from Figure 3.** All additional bands from left or right from a randomly selected protein lysate from the experiment to confirm the proper operation of the immunotransfer device, or positive control lysate chosen according to the antibody's manufacturer recommendation to confirm specificity of the chosen antibody:

- Cldn1: Mouse liver tissue lysates
- Cldn3: Protein lysate from rat's colon or jejunum
- ZO-1 : HepG2
- JAM-A : Protein lysate from rat's colon or jejunum

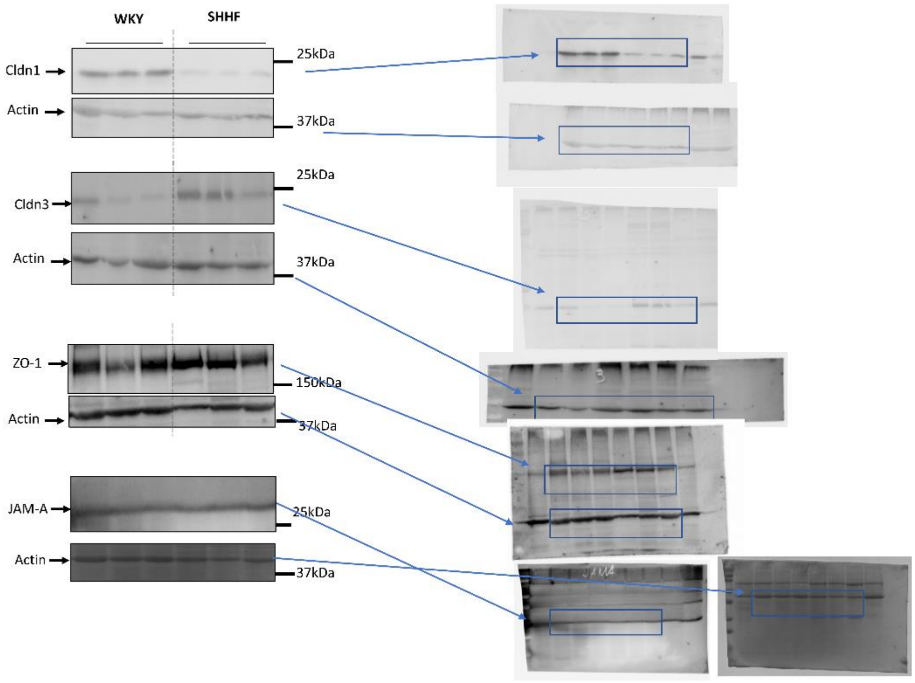

Figure S5. **Uncropped immunoblots from Figure 4.** All additional bands from left or right from a randomly selected protein lysate from the experiment to confirm the proper operation of the immunotransfer device, or positive control lysate chosen according to the antibody's manufacturer recommendation to confirm specificity of the chosen antibody.

- Cldn1: Mouse liver tissue lysates
- Cldn3: Protein lysate from rat's colon or jejunum
- ZO-1 : HepG2
- JAM-A : Protein lysate from rat's colon or jejunum

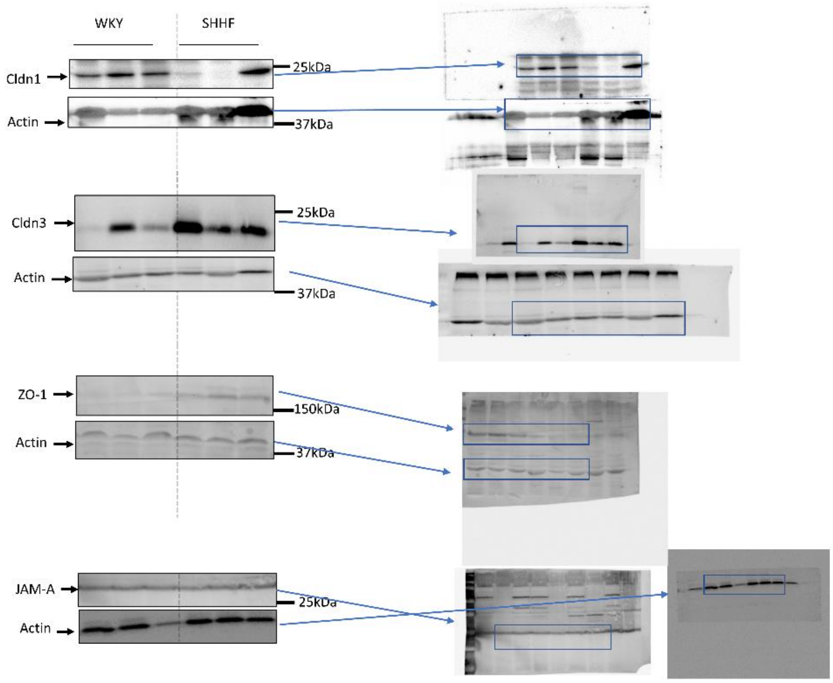

Supplementary Tables

Table S1. List of oligonucleotide primers used for RT-qPCR

**Gene**	**Encoded product**	**Forward (5'-3')**	**Reverse (5'-3')**	**PCR product size [bp]**	**NCBI Accession number**
Cldn 1	Claudin 1	CCGGGCAGATACAGTGCAAA	ACCATCAAGGCTCTGGTTGC	79	NM _031699.2
Cldn 3	Claudin 3	ACCATCACCACTACCAACCG	CTTCCAGCCTAGCAAGCAGA	120	NM _031700.2
TJP 1	Zona Occludens-1	ATCCCACAAGGAGCCATTCC	TAGGGTCACAGTGTGGCAAG	190	NM_001106266.1
F11R	Junctional adhesion molecule A	ACTACTGCGAGGCACAGAAC	CCACGGCTATAGGCAAACCA	163	NM_053796.1
Rpl19	Ribosomal protein L19	CAAGCTGAAGGCAGACAAGGC	CGCTTTCGTGCTTCCTTGGT	87	NM_031103.1

Table S2. List of antibodies used for Western blot analyses.

**Target protein**	**Primary Ab**	**Dilution**	**Secondary Ab**	**Dilution**
Claudin 1	Rabbit polyclonal, Abcam ab180158	1:1000	Goat anti-rabbit, Abcam ab97048-1	1:10,000
Claudin 3	Rabbit polyclonal, Abcam ab15102-50	1:2000	Goat anti-rabbit, Abcam ab97048-1	1:10,000
Zona Occludens-1	Rabbit polyclonal, Abcam ab96587-50	1:500	Goat anti-rabbit, Abcam ab97048-1	1:10,000
Junctional adhesion molecule A	Rabbit polyclonal, Abcam ab180821-100	1:2000	Goat anti-rabbit, Abcam ab97048-1	1:10,000
Beta-Actin	Goat polyclonal, Abcam ab8229-100	1:2000	Donkey anti goat, Abcam ab97107-1	1:20,000
